# Unequivocal imaging of aluminium in human cells and tissues by an improved method using morin

**DOI:** 10.1007/s00418-019-01809-0

**Published:** 2019-08-28

**Authors:** Matthew J. Mold, Manpreet Kumar, William Chu, Christopher Exley

**Affiliations:** 1 Aluminium and Silicon Research Group, The Birchall Centre, Lennard-Jones Laboratories, Keele University, Keele, Staffordshire ST5 5BG UK; 2 School of Life Sciences, Huxley Building, Keele University, Keele, Staffordshire ST5 5BG UK

**Keywords:** Aluminium, Morin, Lumogallion, Fluorescence quenching agents, Familial Alzheimer’s disease, Vaccines

## Abstract

Aluminium is biologically reactive and its ability to potentiate the immune response has driven its inclusion in both veterinary and human vaccines. Consequently, the need for unequivocal visualisation of aluminium in vivo has created a focused research effort to establish fluorescent molecular probes for this purpose. The most commonly used direct fluorescent labels for the detection of aluminium are morin (2′,3,4′,5,7-pentahydroxyflavone) and lumogallion [4-chloro-3-(2,4-dihydroxyphenylazo)-2-hydroxybenzene-1-sulphonic acid]. While the former has gained popularity in the detection of aluminium in plants and predominantly within root tips, the latter boasts greater sensitivity and selectivity for the detection of aluminium in human cells and tissues. Herein, we have developed a simplified morin staining protocol using the autofluorescence quenching agent, Sudan Black B. This modified protocol improves tissue morphology and increases analytical sensitivity, which allows intracellular aluminium to be detected in monocytes and when co-localised with senile plaques in human brain tissue of donors diagnosed with familial Alzheimer’s disease. Overall, our results demonstrate a simple approach to minimise false positives in the use of morin to unequivocally detect aluminium in vivo.

**Electronic supplementary material:**

The online version of this article (10.1007/s00418-019-01809-0) contains supplementary material, which is available to authorized users.

## Introduction

Aluminium is the third most abundant element and the most abundant metal in the Earth’s crust. In spite of its ubiquity, however, aluminium is non-essential to life and its ability to participate in myriad biochemical processes is known to exert toxicity in vivo (Exley 2016). The biologically reactive form of aluminium is its free solvated trivalent metal cation, ${\text{Al}}_{{({\text{aq}})}}^{3 + }$ (Exley and Mold 2015). The increased liberation of the free metal cation in soils by acid rain has shown to be of detriment to the root apical meristem of young seedlings, in which the inhibition of cell elongation and subsequent cell death has been demonstrated (Yamamoto 2019).

The cellular uptake and transport of aluminium in humans are dictated by physicochemical properties including the molecular weight, size and charge of the aluminium species. For example, while low molecular weight neutral complexes of aluminium are predicted to traverse cells via passive transport and enter via passive diffusion (Exley and Mold 2015), particulate forms of the metal ion are transported intracellularly via non-receptor mediated endocytosis (Mold et al. 2014). The latter has relevance for the cellular uptake of aluminium adjuvants that are included in human vaccinations to potentiate and shape the immune response (Reed et al. 2013; Shardlow et al. 2018). Therefore, understanding the mechanisms driving the cellular uptake and fate of aluminium are crucial in the development of both new and existing vaccines (Shardlow et al. 2018).

The fluorophore, lumogallion [4-chloro-3-(2,4-dihydroxyphenylazo)-2-hydroxybenzene-1-sulphonic acid] has demonstrated the unequivocal identification of aluminium in cellular models of vaccination (Mold et al. 2016, 2018a), human sperm cells (Klein et al. 2014) and in inoculated ovine (Asin et al. 2019) and murine tissues (Kashiwagi et al. 2014). Lumogallion has also allowed for the identification of aluminium in animal models in which the metal was identified in the reproductive tissues and brains of Wistar rats exposed to aluminium in their diet (Martinez et al. 2017, 2019). Aluminium has also been visualised using lumogallion in the brains of donors diagnosed with sporadic and familial Alzheimer’s disease (fAD) (Mirza et al. 2017; Mold et al. 2019a), autism spectrum disorder (ASD) (Mold et al. 2018b), multiple sclerosis (MS) (Mold et al. 2018c) and epilepsy (Mold et al. 2019b). Therein, the spatial distribution of aluminium across complex neurological disorders has been visualised in the human brain, allowing for the fate of the metal ion to be elucidated.

Lumogallion acts as a planar tridentate ligand for aluminium coordinating to the metal ion through its two phenolic oxygen ions and the azo group forming aromatic linkages bound through a complex ring structure (Wu et al. 1995). Upon excitation of the fluorophore (500 nm) an orange fluorescence emission (590 nm) is produced. Other physiologically relevant divalent and trivalent metal cations including Ca(II), Cu(II), Mg(II), Zn(II) and Fe(III) (Ren et al. 2001), fail to yield fluorescence in the presence of lumogallion even when prepared as surrogate tissues (Mirza et al. 2016). While competitive equilibria exist between Fe(III) and Al(III), when lumogallion binds to Fe(III), the bond produced is non-fluorescent (Hydes and Liss 1976). As such, high concentrations of Fe(III) have only previously been observed to produce a weaker fluorescence emission for Al(III), that is likely explained by the occlusion of the lumogallion binding site when coordinated to Fe(III) (Hydes and Liss 1976).

While a growing body of evidence demonstrates lumogallion as a sensitive and selective fluorescent molecular probe for the visualisation of aluminium in both cells and tissues, morin (2′,3,4′,5,7-pentahydroxyflavone) continues to predominate studies of aluminium distribution in vivo (Eidi et al. 2015; Chkheidze et al. 2017; Dominguez-Renedo et al. 2019). Unlike lumogallion that coordinates 1:1 with aluminium with high selectivity, morin binds to divalent metal cations including calcium and magnesium, yielding a green fluorescence emission (515 nm) upon excitation at 420 nm, as is observed upon its 3:1 coordination to aluminium (Browne et al. 1990). Such false positives are not encountered using lumogallion (Hydes and Liss 1976; Ren et al. 2001; Mirza et al. 2016). Autofluorescence describes the observation of intrinsic fluorescence in the absence of any added dye or fluorophore. Autofluorescence of human cells and tissues is frequently observed to produce a green fluorescence emission of the non-stained tissue (Klein et al. 2014; Mold et al. 2014; Asin et al. 2019). In contrast to the orange fluorescence signal of lumogallion, morin produces a green fluorescence emission upon binding to aluminium (Browne et al. 1990). Therefore, establishing an autofluorescence background is of critical importance when determining the contribution of the fluorophore, to the overall fluorescence signal observed. Such comparisons are rarely demonstrated in studies using morin for the visualisation of aluminium (Chkheidze et al. 2017).

Herein, we have optimised an improved method for the unequivocal imaging of aluminium in human cells and human brain tissue using morin. Through staining optimisation and selective quenching of background autofluorescence, we unequivocally demonstrate the presence of aluminium using morin, as confirmed by complementary lumogallion staining. Our results highlight notable methodological improvements for the detection of aluminium in vivo utilising morin made achievable by increasing the signal-to-noise ratio of the fluorophore. Taken collectively, our results support autofluorescence quenching as a simple and adaptable approach in visualising directly labelled fluorescent moieties, above background.

## Materials and methods

### T helper 1 cell culture

All chemicals were obtained from Sigma-Aldrich (Poole, UK) unless otherwise stated. T helper 1 (THP-1) cells were obtained from ATCC (TIB-202, LGC Standards, London, UK) and cultured in complete R10 medium containing RPMI 1640 supplemented with GlutaMAX™ containing 25 mM HEPES and 10% v/v heat-inactivated foetal bovine serum (FBS, Certified US Origin, both from Fisher Scientific, Invitrogen, Loughborough, UK). Gentamicin was added at a final concentration of 100 μg/mL from a cell culture certified 10 mg/mL stock solution in ultrapure water, to inhibit microbial growth. Growing cultures were established in canted Tissue Culture (TC) treated and vented T25 cell culture flasks to approximately 1 × 10^6^ cells/mL, prior to sub-culturing into T75 flasks (both from VWR, Corning^®^, Leicestershire, UK). Cells were cultured at 37 °C in a humidified atmosphere containing 5% CO_2_ in a dedicated cell incubator. A Neubauer improved haemocytometer (VWR Marienfeld, Stuttgart, Germany) was used to determine cell density. Cell viability was confirmed by use of the Trypan blue exclusion test in which viable cells excluded the dye from their cytoplasm.

For the assessment of aluminium-based adjuvant (ABA) uptake, THP-1 cells were co-cultured in the absence or presence of an aluminium oxyhydroxide-based adjuvant, Alhydrogel^®^ (2%) (Brenntag Biosector, Frederikssund, Denmark). Native cells were cultured in the presence of R10 medium only. Alhydrogel^®^ was first diluted to 1 mg/mL in a sterile (0.22 μm filtered and autoclaved) 0.9% w/v sodium chloride simulated vaccine diluent, prior to dilution into R10 medium. Cells were plated 1:1 with 100 μL of R10 medium only or with the diluted ABA at a final concentration of 50 μg/mL in 96 well plates (TC treated, VWR, Corning Costar^®^, Leicestershire, UK) and incubated for 24 h at 37 °C (5% CO_2_).

### Human brain tissue

Human brain tissue was supplied following ethical approval (08/MRE09/38 + 5) from the Medical Research Council (MRC) London Neurodegenerative Diseases Brain Bank at Kings College London, UK. Samples of cortex from three donors with familial Alzheimer’s disease (fAD) were provided as paraffin-embedded tissue blocks mounted on Tissue-Tek embedding cassettes (VWR, Sakura^®^ Finetek, Harrisburg, PA, US). Donor A1 is a male donor, aged 47 with Braak/Brain Net Europe (BNE) stage VI pathology (BBN-ID: BBN_16320). Donor A5 is a male donor aged 60 with a Braak stage of at least IV (BBN_13887). Donor A8 is a female donor aged 65 with a presenilin 1 mutation (E280G) and a Braak stage of VI with Lewy bodies (BBN_13813). Early-onset fAD was classified in all donors as described by Mirza and co-workers (Mirza et al. 2017).

### Paraffin embedding of agar-cell blocks

THP-1 cells co-cultured in the absence or presence of Alhydrogel^®^ were pooled across respective cell treatments and pelleted via centrifugation at 800*g* for 10 min, as previously described (Mold et al. 2014). Briefly, cells were re-suspended in 4% w/v paraformaldehyde in 25 mM PIPES, pH 7.4 for 20 min. Fixed cell treatments were washed three times with a 50 mM PIPES buffer adjusted to pH 7.4 with sodium hydroxide pearls, of which 30 μL 5% w/v molten agar was added to the final pellets. HPLC-grade ethanol was used in all procedures whenever required. Agar-cell blocks were gradually dehydrated through an ethanol gradient of 30, 50, 70, 90, 98 and 100% v/v for 20 min, followed by incubation in fresh 100% v/v ethanol, for a further 20 min. Blocks were cleared via transfer into Histo-Clear™ (National Diagnostics, Nottingham, UK) for 40 min, with one change of fresh Histo-Clear™ halfway through. Cleared agar-cell blocks were infiltrated in molten paraffin wax at 60 °C for 40 min and rapidly cast onto Tissue-Tek embedding cassettes (VWR, Sakura^®^ Finetek, Harrisburg, PA, US) on ice, followed by overnight incubation at 4 °C to set the final paraffin-embedded blocks.

### Microtomy

Paraffin-embedded brain tissue and paraffin-embedded agar-cell blocks were cooled on wet ice for 3 h to increase tissue hardness, thereby minimising sample imperfections upon sectioning. All blocks were sectioned by use of a Leica (Wetzlar, Germany) RM2025 rotary microtome using Leica Surgipath DB80LX low profile blades. Ribbons of serially cut sections (i.e. sectioned one after the other) were prepared at a thickness of 5 μm for brain tissue and 2 μm for THP-1 cells. All paraffin-embedded sections were floated out onto ultrapure water (conductivity < 0.067 μS/cm) at 45 °C for 30 s, allowing for gentle tissue expansion. Sections for brain tissue and THP-1 cells were caught on numbered electrostatically charged SuperFrost^®^ Plus glass slides (Thermo Fisher Scientific, Loughborough, UK), respectively. Sections were subsequently stored vertically and allowed to dry at ambient temperature overnight. For paraffin-embedded brain tissue only, sections were incubated in a paraffin slide heater at 60 °C for 20 min to remove excess paraffin and were allowed to cool to ambient temperature, prior to staining.

### Deparaffinisation and rehydration procedures

Directly adjacent and numbered serial THP-1 cell or brain tissue sections were prepared as separate sets, to allow for their analysis via morin, lumogallion or autofluorescence quenching, respectively. Solvents for deparaffinisation and rehydration of paraffin-embedded sections were prepared at a final volume of 250 mL in glass staining dishes. All brain tissue and THP-1 cell sections were deparaffinised with gentle agitation via immersion into Histo-Clear™ for 3 min. To ensure that any residual paraffin was removed, sections were immersed in fresh Histo-Clear™ for a further minute. Sections were gradually rehydrated through an ethanol gradient via transfer for 1 min into 100, 95, 70, 50 and 30% v/v ethanol. Sections were finally immersed in ultrapure water and agitated continuously for 35 s. A PAP pen was used to create a hydrophobic barrier around rehydrated sections, allowing for low reagent volumes (200 μL) to be used via staining in humidity chambers. Sections were subsequently stained and mounted according to the following staining procedures.

### Lumogallion staining

Lumogallion [4-chloro-3-(2,4-dihydroxyphenylazo)-2-hydroxybenzene-1-sulphonic acid] (Tokyo Chemical Industry, Oxford, UK) was prepared at 1 mM via dilution into 0.22 μm filtered 50 mM PIPES buffer, pH 7.4. Deparaffinised and rehydrated THP-1 cell or brain serial sections were stained away from light for 45 min at ambient temperature with either lumogallion or 50 mM PIPES buffer only, of which the latter acted as a control for autofluorescence. Following staining, sections were subsequently washed six times with 200 μL aliquots of 50 mM PIPES buffer, prior to a 30 s rinse in ultrapure water. Mounting of stained sections was performed using glass coverslips using the aqueous mounting medium Fluoromount™ for all brain tissue sections and the aqueous mounting medium, ProLong^®^ Gold Antifade Reagent with 4′,6-diamidino-2-phenylindole, dihydrochloride (DAPI) (Life Technologies, Fisher Scientific, Loughborough, UK) for THP-1 cell sections. The latter was used to highlight THP-1 cell nuclei. All sections were incubated overnight at 4 °C away from light allowing for the respective polymers to harden, prior to analysis via fluorescence microscopy.

### Sudan Black B as an autofluorescence quenching agent of lumogallion-stained human brain tissue sections

Sudan Black B (SBB) was prepared as an autofluorescence quenching agent via dilution in 70% v/v ethanol and was centrifuged for 5 min at 8000*g*, immediately prior to use. Prior to mounting, lumogallion-stained brain tissue sections were post-incubated with 0.01 or 0.10% w/v SBB, washed six times with 200 μL aliquots of 70% v/v ethanol and rinsed for 30 s in ultrapure water. Sections were subsequently mounted using Fluoromount™ and were incubated overnight at 4 °C away from light, prior to analysis via fluorescence microscopy.

### Shaw’s morin staining

Conventional Shaw’s morin staining was performed as previously reported (Shaw and Petrik 2009) and is outlined in Table 1. Briefly, morin was prepared at 0.2% w/v in 85% v/v ethanol, containing 0.5% v/v acetic acid. Rehydrated THP-1 or human brain tissue sections were twice incubated in fresh solutions of 0.22 μm filtered phosphate buffered saline (PBS), pH 7.0, for 5 min. Sections were subsequently acidified in 1% v/v HCl for 10 min, followed by two 5 min washes in ultrapure water. Morin staining was performed in humidity chambers via the addition of 200 μL of the fluorophore, of which sections were incubated for 10 min. Following staining, sections were twice rinsed via immersion in ultrapure for 5 min, sequentially dehydrated through 70, 90 and 100% v/v ethanol and cleared with xylene (via full immersion in each solvent for approximately 30 s). Cleared and stained sections were immediately mounted using glass coverslips with the DPX-based mounting medium, Omnimount™ (National Diagnostics, Nottingham, UK). Stained and mounted sections were allowed to cure fully and were sealed with clear nail varnish, prior to analysis via fluorescence microscopy.

**Table 1:** A comparison between protocols and durations for Shaw’s morin staining and an optimised morin staining protocol for deparaffinised and rehydrated cell and brain tissue sections (Shaw and Petrik 2009)

Shaw’s morin staining			Optimised morin staining		
Steps	Reagent	Time	Steps	Reagent	Time
1.	PBS, pH 7.0	5 min	1.	0.2% w/v morin, 85% v/v ethanol	30 min
2.	PBS, pH 7.0	5 min	2.	85% v/v ethanol	30 s
3.	1% v/v HCl	10 min	3.	Ultrapure water	35 s
4.	Ultrapure water	5 min	4.	0.3% w/v Sudan Black B, 70% v/v ethanol	10 min
5.	Ultrapure water	5 min	5.	70% v/v ethanol	30 s
6.	0.2% w/v morin, 85% v/v ethanol, 0.5% v/v acetic acid	10 min	6.	Ultrapure water	30 s
7.	Ultrapure water	5 min		Total Time	~ 42 min
8.	Ultrapure water	5 min			
9.	70% v/v ethanol	30 s			
10.	90% v/v ethanol	30 s			
11.	100% v/v ethanol	30 s			
12.	Xylene	30 s			
	Total time	~ 52 min			

### Optimised morin staining using Sudan Black B

A comparison of staining protocols for conventional Shaw’s morin staining (Shaw and Petrik 2009) versus an optimised morin staining protocol developed herein, is given in Table 1. Shaw’s morin solution omitting the addition of acetic acid was added to rehydrated tissue sections for 30 min in humidity chambers. Following morin staining, tissue sections were washed six times with 200 μL aliquots of 85% v/v ethanol and rinsed in ultrapure water for 35 s. Tissue sections were subsequently incubated for 10 min in 0.3% w/v SBB in 70% v/v ethanol, washed six times with 200 μL aliquots of 70% v/v ethanol and rinsed for 30 s in ultrapure water (Table 1). THP-1 cell sections prepared in this way were mounted with ProLong Gold antifade reagent with DAPI under glass coverslips (Life Technologies, Fisher Scientific, Loughborough, UK). Brain tissue sections were mounted with Fluoromount™ and all sections were incubated at 4 °C overnight, prior to their analysis via fluorescence microscopy.

### Ethylenediaminetetraacetic acid as an autofluorescence quenching agent

Non-specific staining of morin attributed to the binding of magnesium and calcium was minimised via pre-soaking rehydrated THP-1 cell sections in 250.0 mL of 0.22 μm filtered 5 mM ethylenediaminetetraacetic acid (Na_2_EDTA) for 10 min (Browne et al. 1990). Sections were subsequently washed in ultrapure water for 35 s, prior to staining in modified Shaw’s morin omitting the addition of acetic acid for the reduced duration of 10 min. Sections were subsequently rinsed for 30 s in 85% v/v followed by a 35 s rinse in ultrapure water, prior to mounting with ProLong Gold antifade reagent with DAPI (Life Technologies, Fisher Scientific, Loughborough, UK).

### Fluorescence microscopy

Human brain and THP-1 cell sections were analysed using an Olympus BX50 fluorescence microscope equipped with a vertical illuminator and BX-FLA reflected fluorescent light attachment (mercury source). Fluorescence micrographs were obtained by use of a X 40 Plan-Fluorite objective or under a low autofluorescence immersion oil (Olympus immersion oil, type-F) under a X 100 Plan-Fluorite objective (both from Olympus, Southend-on-Sea, UK), respectively. Lumogallion (orange) and morin (green) fluorescence were visualised using a U-MNIB3 fluorescence filter cube (bandpass *λ*_ex_: 470–495 nm, dichromatic mirror: 505 nm, longpass *λ*_em_: 510 nm) and U-MWBV2 cube (bandpass *λ*_ex_: 400–440 nm, dichromatic mirror: 455 nm, longpass *λ*_em_: 475 nm), respectively. DAPI staining of cell nuclei (blue) was visualised by use of a U-MWU2 (bandpass *λ*_ex_: 330–385 nm, dichromatic mirror: 400 nm, longpass *λ*_em_: 420 nm) filter cube. All filter cubes were purchased from Olympus (Southend-on-Sea, UK). Light transmission and exposure values were fixed across treatment conditions and images captured using a ColorView III CCD camera using the CellD software suite (Olympus, Soft Imaging Solutions, SiS, GmbH, Münster, Germany). Merging of bright-field and fluorescence channels was performed using Photoshop (Adobe Systems Inc., Palo Alto, CA, US).

## Results

### Post-staining with Sudan Black B (SBB) aids in the visualisation of intracellular aluminium adjuvant in T helper 1 monocytes

**Fig. 1:**
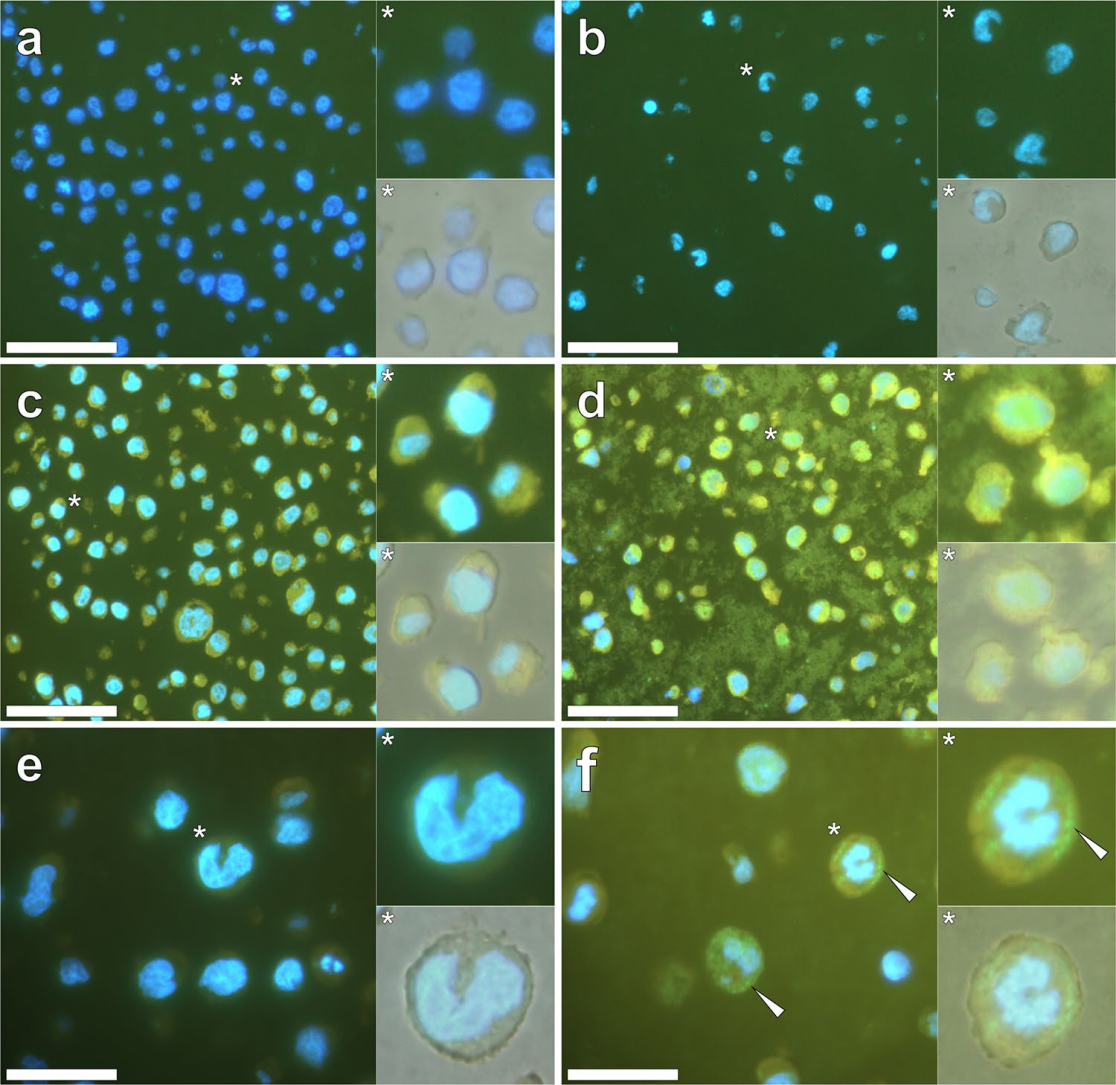
Optimisation of morin staining for the detection of intracellular Alhydrogel^®^ in sectioned THP-1 cells performed in the absence and presence of the autofluorescence quenching agent, Sudan Black B (SBB). **a** Native THP-1 cells stained with 0.3% w/v SBB only (10 min), **c** 0.2% w/v morin only (30 min) or **e** stained with morin and post-stained with SBB, respectively. **b**, **d** and **f** THP-1 cells co-cultured with 50 μg/mL Alhydrogel^®^ and stained as with native THP-1 cells, respectively. All sections were mounted with ProLong Gold antifade reagent with DAPI and fluorescence micrographs depict morin staining (green) (U-MWBV2, longpass *λ*_em_: 475 nm) with DAPI fluorescence (blue) (U-MWU2, longpass *λ*_em_: 420 nm) overlaid. Magnified inserts are depicted with the bright-field channel merged in the lower panels. White arrows highlight distinguishable intracellular particulates of the adjuvant. Scale bars: (**a**–**d**): 50 μm, (**e**, **f**): 20 μm

Native T helper 1 (THP-1) cells cultured for 24 h in the absence (Fig. 1a), or presence (Fig. 1b) of 50 μg/mL of an aluminium oxyhydroxide-based Alhydrogel^®^ adjuvant and stained with 0.3% w/v Sudan Black B (SBB) only, produced no detectable morin fluorescence (U-MWBV2, longpass *λ*_em_: 475 nm). While SBB was found to quench cellular autofluorescence, positive DAPI staining of post-SBB stained THP-1 cell nuclei remained readily detected by a blue fluorescence emission, in all treatments (Fig. 1). Morin staining only in treatments containing native THP-1 cells (Fig. 1c) or those co-cultured with Alhydrogel^®^ (Fig. 1d), both revealed a uniform green fluorescence emission contained within cell cytosol. As such, while the latter cell treatments clearly depicted extracellular adjuvant (Fig. 1d), its internalisation versus native cells (Fig. 1c) was rendered indistinguishable.

Post-incubation of morin-stained native THP-1 cells with SBB, quenched autofluorescence as evidenced by a diminished fluorescence intensity within the cell cytoplasm (Fig. 1e). Interestingly, under identical staining conditions THP-1 cells co-cultured with 50 μg/mL Alhydrogel^®^ for 24 h, revealed punctate intracellular fluorescence (Fig. 1f). Weak intracellular fluorescence was initially observed (see Supplementary Fig. 1) under illumination settings typically used for the visualisation of morin fluorescence (excitation: 470–495 nm, dichromatic mirror: 505 nm, longpass emission: 510 nm). Illumination under an alternative morin filter cube increased the intensity of the intracellular fluorescence signal (U-MWBV2, excitation: 400–440 nm, dichromatic mirror: 455 nm, longpass emission: 475 nm) and was, therefore, used in all post-SBB staining experiments.

Therein morin-stained THP-1 cells post-labelled with SBB demonstrated intracellular particulates with an outer diameter of 0.97 ± 0.13 μm (mean ± SD, *n* = 30) that were found exclusively within the cytoplasm of THP-1 cells (Fig. 1f). Furthermore, extracellular fluorescence was quenched with minimal extracellular particulate material observed across THP-1 cell sections (Fig. 1f). Conventional Shaw’s morin staining of native THP-1 cells prepared in the absence of added adjuvant produced a yellow fluorescence emission in cell cytosol (see Supplementary Fig. 2). Similarly, cells co-cultured in the presence of 50 μg/mL Alhydrogel^®^ produced a similar yellow fluorescence emission, thereby hindering the detection of intracellular particulates of the adjuvant. Such was not resolved even when cells were illuminated under the modified morin filter set, as used in the optimised SBB-staining experiments.

### Metal chelation using ethylenediaminetetraacetic acid (EDTA) as a fluorescence quenching agent

**Fig. 2:**
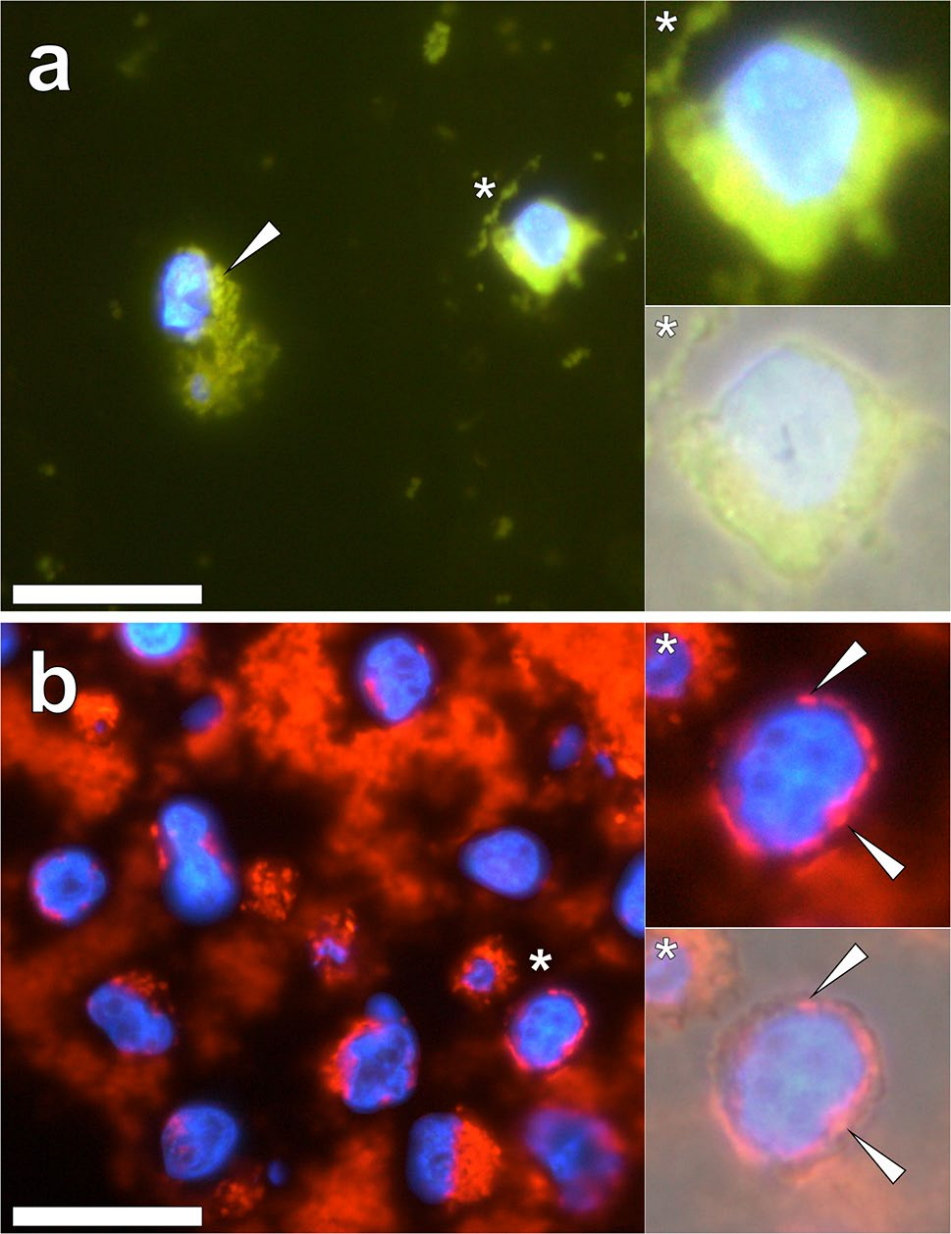
Morin (green) and lumogallion (orange) fluorescence of THP-1 cells co-cultured with 50 μg/mL of an aluminium oxyhydroxide-based Alhydrogel^®^ adjuvant. **a** Deparaffinised and rehydrated 5 μm cell sections pre-treated with Na_2_EDTA at 5 mM for 10 min, stained in 0.2% w/v morin in 85% v/v ethanol for 10 min and viewed under a U-MNIB3 (longpass *λ*_em_: 510 nm) fluorescence filter cube. **b** Cell sections stained with 1 mM lumogallion for 45 min and viewed under a modified U-MNIB3 (single bandpass *λ*_em_: 570–610 nm) filter cube. Sections were mounted with ProLong Gold antifade reagent with DAPI (Fisher Scientific, UK), highlighting the presence of cell nuclei via a blue fluorescence emission captured under a U-MWU2 filter channel (longpass *λ*_em_: 420 nm). Lower magnified inserts (asterisks) depict the merging of fluorescence with the bright-field channel. White arrows indicate the presence of intracellular particulates of the adjuvant. Scale bars: 20 μm

Further attempts to reduce intrinsic cellular autofluorescence of morin-stained THP-1 cells were made via their pre-treatment using the hexadentate ligand, ethylenediaminetetraacetic acid (EDTA). THP-1 cells co-cultured with 50 μg/mL Alhydrogel^®^, pre-treated with EDTA and stained with morin revealed a green fluorescence emission contained within cell cytosol (Fig. 2a). Therein, punctate fluorescence was observed, however the identification of intracellular particulates was at times hindered owing to an intense fluorescence emission masking intracellular adjuvant upon staining. Green fluorescence was also noted within the cytoplasm of native THP-1 cells stained in an identical manner, thereby accounting for their high fluorescence background (see Supplementary Fig. 3).

Identical THP-1 cell treatments co-cultured with 50 μg/mL Alhydrogel^®^ and stained with lumogallion revealed an intense intracellular punctate orange (single bandpass *λ*_em_: 570–610 nm) fluorescence emission (Fig. 2b). Therein, discrete particles with an outer diameter of approximately 1 μm were found contained within the cytoplasm of THP-1 cells, as confirmed by merging of the bright-field channel. Counter-staining with DAPI confirmed the absence of intracellular particulate material within cell nuclei (Fig. 2b). Native THP-1 cells stained with lumogallion in an identical manner revealed a weak brown/orange fluorescence emission that was clearly distinguishable against internalised lumogallion-reactive particulates (see Supplementary Fig. 3).

### Conventional morin staining of human brain tissue sections

**Fig. 3:**
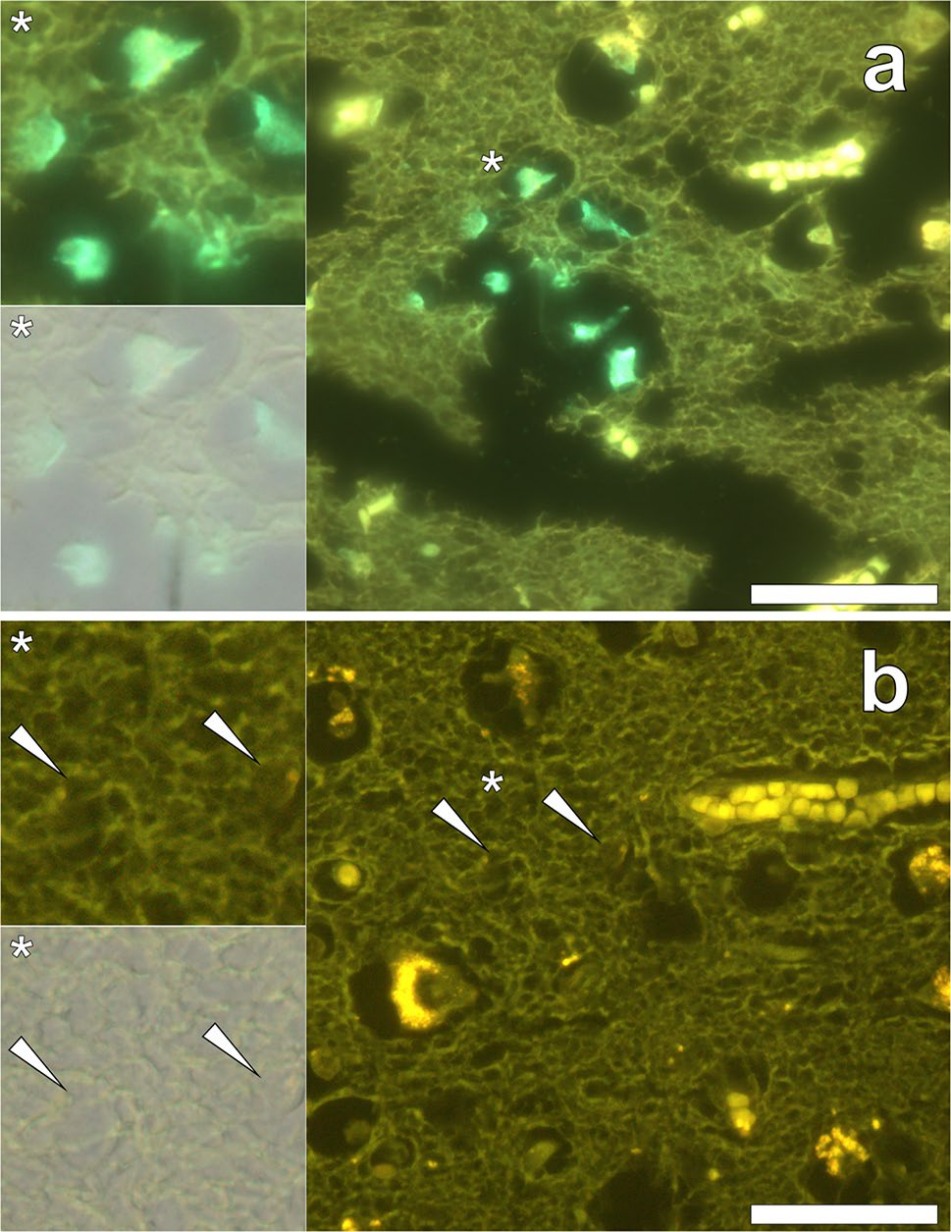
Conventional Shaw’s morin and lumogallion staining of the parietal lobe of a donor (A1) diagnosed with familial Alzheimer’s disease (fAD). **a** Shaw’s morin staining of sectioned (5 μm) human brain tissue revealing a positive green fluorescence emission as viewed under a U-MWBV2 fluorescence filter channel (longpass *λ*_em_: 475 nm). **b** Lumogallion staining (U-MNIB3, longpass *λ*_em_: 510 nm) of an adjacent serial section, demonstrating a lack of positive aluminium staining (orange) in the same regions as those highlighted using morin fluorescence, as indicated by white arrows. Sections were mounted with the resinous mounting media Omnimount™ (National Diagnostics, UK). Magnified inserts are depicted (asterisks) of which the lower panels show fluorescence merged with the bright-field channel. Scale bars: 50 μm

Tissue sections of donors diagnosed with familial Alzheimer’s disease (fAD) were stained for the presence of aluminium using conventional Shaw’s morin staining. Therein, a positive green intracellular fluorescence emission was observed within astrocytic and neuronal-like cells in grey matter of the parietal lobe of a 47-year-old male (donor A1), diagnosed with early-onset fAD (Fig. 3a). Occasional intracellular accumulations of lipofuscin were also noted within neuronal-like cells via punctate deposits, highlighted via a yellow fluorescence emission (Fig. 3a). Tissue integrity of conventional Shaw’s morin-stained sections was found to be poor, in which damage appearing as tears and lost tissue was frequently observed.

Lumogallion staining of the adjacent section (Fig. 3b) revealed the presence of intracellular lipofuscin deposits that were identified via punctate yellow fluorescence, within neuronal-like cells. Interestingly, a positive (orange) fluorescence emission for lumogallion-reactive aluminium was not identified within adjacent serially sectioned cells, demonstrating the absence of the metal ion (Fig. 3b). Therefore, these results identified false positives in utilising conventional Shaw’s morin staining for the identification of aluminium in human brain tissue.

### Post-staining with Sudan Black B (SBB) aids in the visualisation of aluminium in human brain tissue

**Fig. 4:**
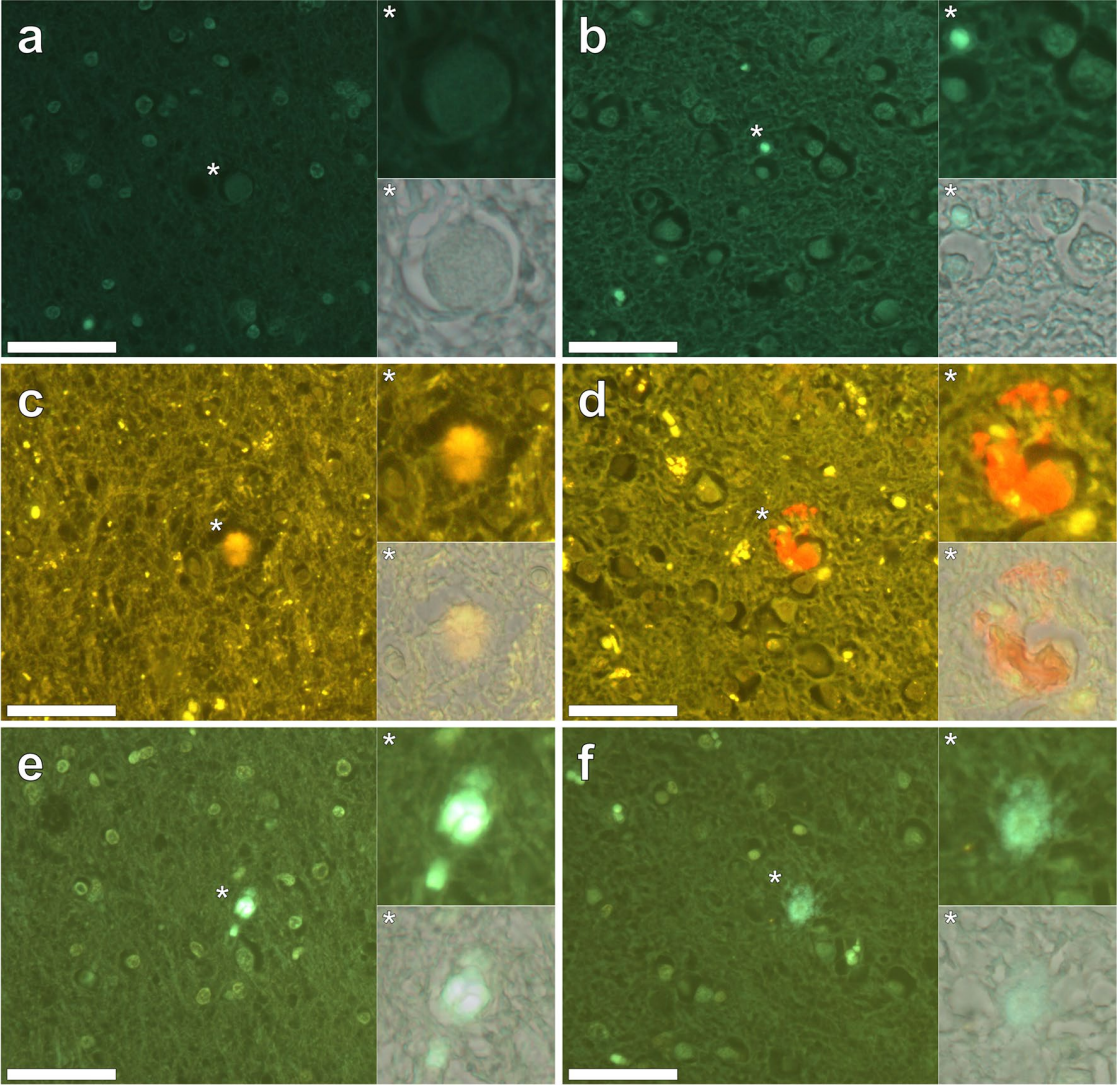
Morin and lumogallion staining of adjacent serial sections from the frontal (**a**, **c** and **e**) and occipital (**b**, **d** and **f**) lobes of a donor (A5) diagnosed with familial Alzheimer’s disease (fAD). **a**, **b** 0.3% w/v Sudan Black B (SBB) staining (10 min), **c**, **d** 1 mM lumogallion staining (45 min) and **e**, **f** 0.2% w/v morin staining (30 min) followed by SBB autofluorescence quenching (10 min). Lumogallion (orange) (U-MNIB3, longpass *λ*_em_: 510 nm) and morin fluorescence (green) (U-MWBV2, longpass *λ*_em_: 475 nm) were identified in identical regions when prepared and stained as adjacent (5 μm) serial sections. Magnified inserts are highlighted (asterisks), of which the lower panels show merging of the bright-field image with the respective morin (**a**, **b**, **e** and **f**) and lumogallion (**c** and **d**) fluorescence channels. Scale bars: 50 μm

In order to assess the efficacy of morin for the positive detection of aluminium in human brain tissue, optimised morin staining was performed in an identical manner to THP-1 cells post-stained with SBB. Staining of the frontal and occipital cortex of a 60-year-old male donor (A5) diagnosed with familial Alzheimer’s disease (fAD) with 0.3% w/v SBB only, produced an overall weak green fluorescence emission. Use of a modified morin filter set (longpass *λ*_em_: 475 nm) revealed an occasional blue-green fluorescence emission noted within lipofuscin-like deposits in white (Fig. 4a) and grey (Fig. 4b) matter of the frontal and occipital lobes, respectively.

Lumogallion staining of adjacent serial sections identified widespread lipofuscin deposition as identified via punctate yellow fluorescence concurrent with intracellular fluorescence in SBB only stained sections (Fig. 4c, d). Therein, a positive orange fluorescence emission indicative of lumogallion-reactive aluminium was noted within senile plaque-like structures (Fig. 4c) and within cortical neurons (Fig. 4d). Optimised morin staining and SBB fluorescence quenching of adjacent serial sections revealed positive green morin fluorescence, thereby indicating the presence of aluminium within identical regions of the frontal (Fig. 4e) and occipital (Fig. 4f) lobes, respectively.

In order to assess the efficacy of SBB in quenching lipofuscin fluorescence of non-stained and lumogallion-stained tissue sections, a post-incubation step utilising SBB was introduced. Autofluorescence of the parietal cortex of a 65-year-old female donor (A8) diagnosed with fAD, revealed a diffuse cortical senile-plaque approximately 25 μm in diameter, as highlighted via a green fluorescence emission (see Supplementary Fig. 4a). Yellow punctate fluorescent pigments of lipofuscin were readily identified and their presence was also noted on lumogallion-stained sections (Supplementary Fig. 4b). Lumogallion staining of the identical senile plaque in a serial section revealed an orange fluorescence emission indicative of the presence of aluminium. Post-staining of adjacent lumogallion-stained sections with 0.01 and 0.10% w/v SBB quenched lipofuscin fluorescence, successfully masking its appearance (Supplementary Fig. 4c and d). At the highest SBB concentration of 0.10%, autofluorescence was near-abolished concomitant with reduced fluorescence intensity of positive lumogallion fluorescence.

## Discussion

We have optimised the use of morin to allow for the unequivocal detection of aluminium in human cells and brain tissue. Owing to a bright excitation wavelength at approximately 400 nm for mercury sources of illumination, a more intense fluorescence emission was observed for morin complexed to aluminium under an Olympus U-MWBV2 fluorescence filter cube (Mold et al. 2018a). Such acted as a simple means of increasing the fluorescence intensity of morin-stained human cells and tissue sections.

While morin reacts with aluminium to produce a fluorescent compound, it is also prone to false positives through non-preferential binding to physiologically relevant metal cations, including magnesium and calcium (Browne et al. 1990). Furthermore, the green fluorescence emission (515 nm) of the fluorophore upon complexation to aluminium (Browne et al. 1990) occurs at wavelengths similar to intrinsic autofluorescence produced upon excitation of non-stained human cells and tissues (Mold et al. 2014; Mirza et al. 2017). Such spectral overlay hinders the identification of a positive green fluorescence emission above a green autofluorescence background.

Such interferences have been overcome in the present study by post-incubating morin-stained cells and tissues with the fluorescence quenching agent Sudan Black B (SBB) (Table 1). SBB is a fat-soluble and hence, lysochrome-based, diazo dye that stains triglycerides and lipids in histological sections (Sun et al. 2011; Erben et al. 2016). Importantly, SBB is able to quench intrinsic autofluorescence of non-stained specimens through its binding to intrinsically fluorescent proteins and lipids, producing an opaque labelling (Schnell et al. 1999). Herein, the ability of SBB to absorb the energy released from intrinsically fluorescent tissue components in the form of photons was exploited in an improved staining method for morin, with the overall effect of reducing autofluorescence (Schnell et al. 1999).

SBB prepared at 0.3% w/v in 70% ethanol was found optimal for the post-quenching of autofluorescence in monocytic THP-1 cells co-cultured with an aluminium oxyhydroxide-based vaccine adjuvant, Alhydrogel^®^. Quenching of autofluorescence was only necessary when using morin to detect intracellular particulates of the adjuvant versus the unequivocal detection offered by the fluorophore, lumogallion (Mold et al. 2014). Intracellular particulates of outer diameter 0.97 ± 0.13 μm (mean ± SD, *n* = 30) were observed in THP-1 cells, in agreement with our previous studies of adjuvant uptake in this cell line of 0.96 ± 0.19 μm (mean ± SD, *n* = 340) (Mold et al. 2016).

Alhydrogel^®^ is the most commonly used aluminium-based adjuvant (ABA) yet its inclusion in vaccines has been linked to the condition of macrophagic myofasciitis (MMF) in humans, forming granulomatous lesions (Gherardi and Authier 2012; Shardlow et al. 2018). Therefore, the unequivocal tracing of ABAs in biological systems is of critical importance in the design of safe and effective vaccines targeting disease. However, studies tracing the presence of Alhydrogel^®^ containing vaccines following their intramuscular administration in MMF, have continued to use morin in the absence of suitable autofluorescence controls (Chkheidze et al. 2017). These interferences could be easily overcome via the use of fluorescence quenching agents including SBB as optimised in this work. In a recent study highlighting morin as a sensitive and selective stain for the visualisation of aluminium in MMF tissues, such was substantiated by the use of the ligand, EDTA (Chkheidze et al. 2017). Therein, post-incubation of morin-stained sections with EDTA abolished morin fluorescence.

While perhaps unsurprising that EDTA was capable of competitively chelating aluminium resulting in a reduction of the fluorescence intensity, this approach did not prove nor disprove that the fluorescence observed was as a result of interfering metal ions and or background autofluorescence (Chkheidze et al. 2017). Such may be further compounded when using positive fluorescence emission from morin-stained cells to quantitatively assess migration of aluminium into the central nervous system (Inohana et al. 2018). Therefore, experiments utilising morin for the detection of aluminium must ensure that background autofluorescence is suitably low or quenched and non-specific binding and false positives are accounted for.

Herein, we have adapted the use of EDTA as a pre-treatment phase during staining which was found to increase the contrast of internalised ABA particulates, though not as effectively as post-staining with SBB. EDTA preferentially binds to divalent metal ions and its use prior to staining would allow for interfering divalent metal ions to be chelated and removed, prior to staining for aluminium (Yokel 1994). It is of note, however, that EDTA coordinates to aluminium and while its binding affinity is lower versus trivalent ion coordinators including desferrioxamine, both ligands have been historically used to successfully chelate aluminium (Alfrey et al. 1976; Wills and Savory 1989; Yokel 1994). Overall, chelation using EDTA as a pre-treatment to staining would remove aluminium from the target tissues of interest resulting in the potential for false negatives when used with morin for the detection of aluminium.

SBB allowed for the unequivocal determination of aluminium using morin complemented with lumogallion staining, in human brain tissue of donors diagnosed with familial Alzheimer’s disease (fAD). This early-onset form of Alzheimer’s disease is rare and mutations typically found in the amyloid precursor protein (APP) and presenilin (PSEN) genes exacerbate the formation of the neurotoxic and pathologically related peptide, amyloid-β_42_ (Goate et al. 1991; Sherrington et al. 1995; Pimplikar 2009). Subsequently, the peptide undergoes self-aggregation forming senile plaques that deposit in fAD tissues that are sinks for metal ions including aluminium and iron (Khan et al. 2006; Yumoto et al. 2009; Exley and House 2011; Mirza et al. 2017; Mujika et al. 2018). Therefore, direct fluorescent labels allowing for the unequivocal detection of aluminium in senile plaques and related neuropathological hallmarks of Alzheimer’s disease are of pertinent interest (Mirza et al. 2017).

Conventional Shaw’s morin staining damaged fAD donor tissue sections as a result of the HCl pre-rinse. Such was implemented in the original protocol to prevent interferences in the binding of calcium and magnesium to morin (Shaw and Petrik 2009). Furthermore, positive intracellular fluorescence indicating the presence of aluminium in glial and non-neuronal cells in morin-stained sections herein was not detectable upon adjacent lumogallion-stained sections. This highlighted false positives in the use of Shaw’s morin staining in identifying aluminium in human brain tissue. Tissue sections of fAD donors additionally revealed lipofuscin in both stained and non-stained sections. Lipofuscin is an ageing-associated pigment that accumulates in neurones and fluoresces across a broad spectral range (Schnell et al. 1999). SBB has been shown to be effective in quenching lipofuscin fluorescence in even the thickest (250–350 μm) of human spinal cord and brain tissue sections in a procedure called Brain Lipids and Aldehyde Quench (BLAQ) (Kupferschmidt et al. 2015).

Herein, SBB successfully quenched lipofuscin and background autofluorescence allowing for a marked improvement in the identification of positive morin fluorescence. This was made possible through improvements to the signal-to-noise ratio of the fluorophore. Use of this optimised protocol removed the need for pre-treatment measures to remove interfering metal ions with the additional benefit of omitting the use of glacial acetic acid from morin staining solutions (Shaw and Petrik 2009). The latter increases the acidity of the morin staining solution increasing the likelihood of aluminium leaching from stained tissues in the form of ${\text{Al}}_{{({\text{aq}})}}^{3 + }$ (Exley 1996; Mold et al. 2014). Importantly, lumogallion was capable of unequivocally identifying aluminium in human cells and tissues without the need for additional pre-treatment steps nor post-staining with SBB. Use of this improved method for morin allowed for intracellular aluminium to be detected in cortical neurons and extracellular aluminium to be detected in senile plaques in human brain tissue of donors diagnosed with fAD, as has now been demonstrated with lumogallion in a number of recent studies (Mirza et al. 2017; Mold et al. 2014, 2016, 2018a, b, c, 2019a, b). In summary, we have developed a both simplified and optimised morin staining protocol that allowed for the unequivocal detection of aluminium in both human cells and brain tissue. These methods are immediately applicable to the use of both new and existing fluorophores for the unequivocal detection of aluminium in vivo.

## Electronic supplementary material

Below is the link to the electronic supplementary material.

Supplementary material 1 (DOCX 10423 kb)
