# Supplementary material for: Unequivocal imaging of aluminium in human cells and tissues by an improved method using morin
Source: Histochem Cell Biol. 2019 Aug 28;152(6):453–63. doi: 10.1007/s00418-019-01809-0 (PMC6881412; doi:10.1007/s00418-019-01809-0)
Supplement: Supplementary file 1 — Supplementary material 1 (DOCX 10423 kb) [file 418_2019_1809_MOESM1_ESM.docx]

**Supplementary Material**

**Title:**

Unequivocal imaging of aluminium in human cells and tissues by an improved method for morin

**Authors**:

Matthew J. Mold*, Manpreet Kumar, William Chu & Christopher Exley.

**Postal addresses:**

The Birchall Centre, Lennard-Jones Laboratories, Keele University, Keele, Staffordshire, ST5 5BG, UK (MJM & CE). School of Life Sciences, Huxley Building, Keele University, Keele, Staffordshire, ST5 5BG, UK (MK & WC).

**Corresponding author*:**

Matthew Mold PhD MRSB (ORCID ID: 0000-0002-4616-6204)

Research Fellow

Aluminium and Silicon Research Group

The Birchall Centre, Lennard-Jones Laboratories,

Keele University, Staffordshire, ST5 5BG, UK

Tel: 44 (0) 1782 733508; Email: [m.j.mold@keele.ac.uk](mailto:m.j.mold@keele.ac.uk)

**Acquisition of morin fluorescence using an alternative fluorescence filter cube aids in the visualisation of intracellular aluminium**

**
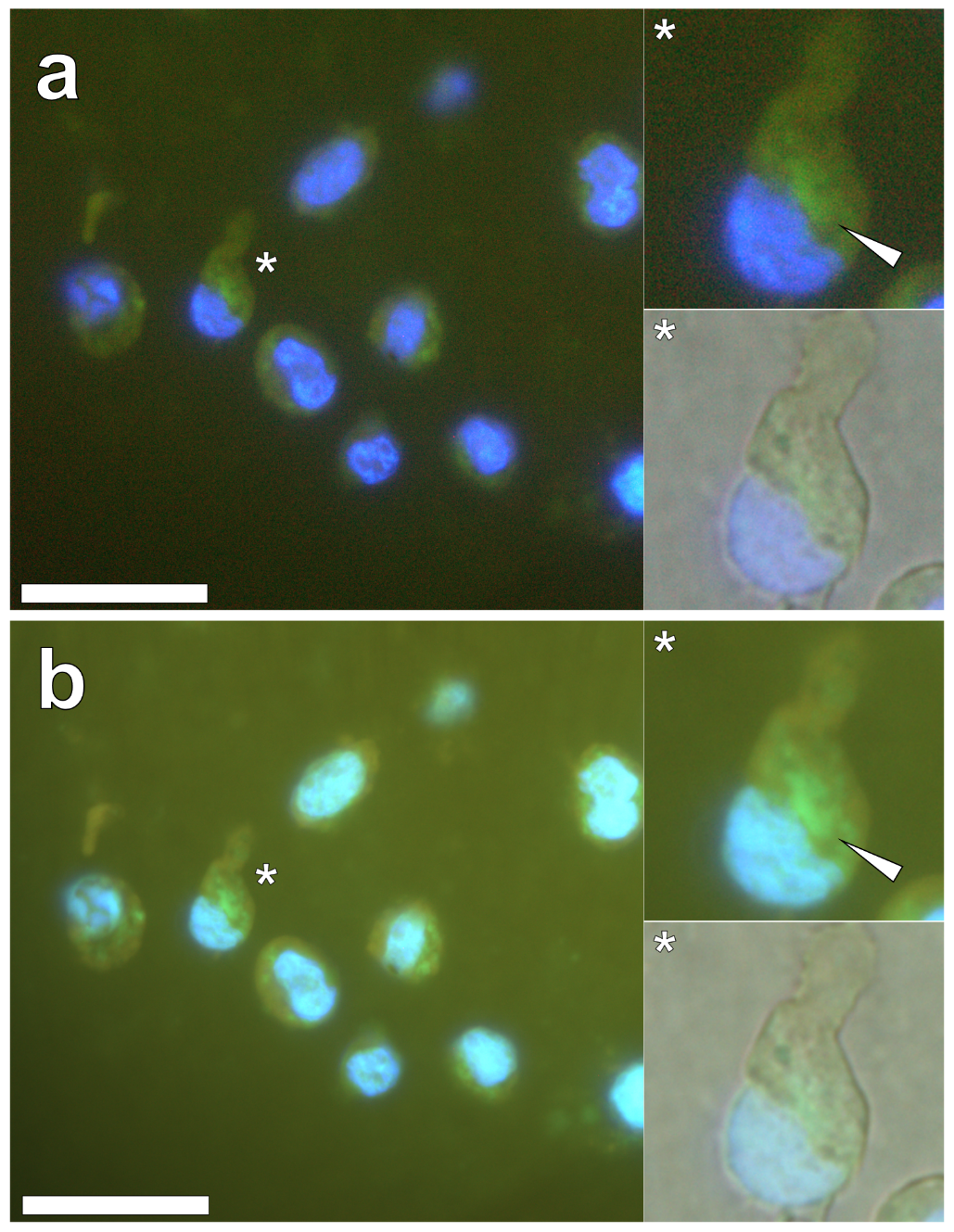
**

**Supplementary Fig. 1** T helper 1 (THP-1) monocytes co-cultured with 50μg/mL of the aluminium oxyhydroxide adjuvant, Alhydrogel^®^ for 24h and stained for the presence of aluminium using morin. Sectioned (5μm) THP-1 cells were stained with 0.2% *w/v* morin in 85% *v/v* ethanol for 30 min, rinsed and post-stained for 10 min in Sudan Black B (SBB). Washed sections were mounted with ProLong Gold antifade reagent with DAPI (Life Technologies, UK). Cells analysed under a U-MNIB3 (longpass λ_em_: 510nm) **(a)** fluorescence filter cube are depicted versus heightened intracellular fluorescence (white arrows) observed under a U-MWBV2 **(b)** (longpass λ_em_: 475nm) cube (both from Olympus, UK), with DAPI fluorescence (blue) (U-MWU2, longpass λ_em_: 420nm) overlaid. Scale bars: 20μm.

**Conventional morin staining of THP-1 cells co-cultured in the absence and presence of 50μg/mL of an aluminium oxyhydroxide based Alhydrogel^®^ adjuvant**

**
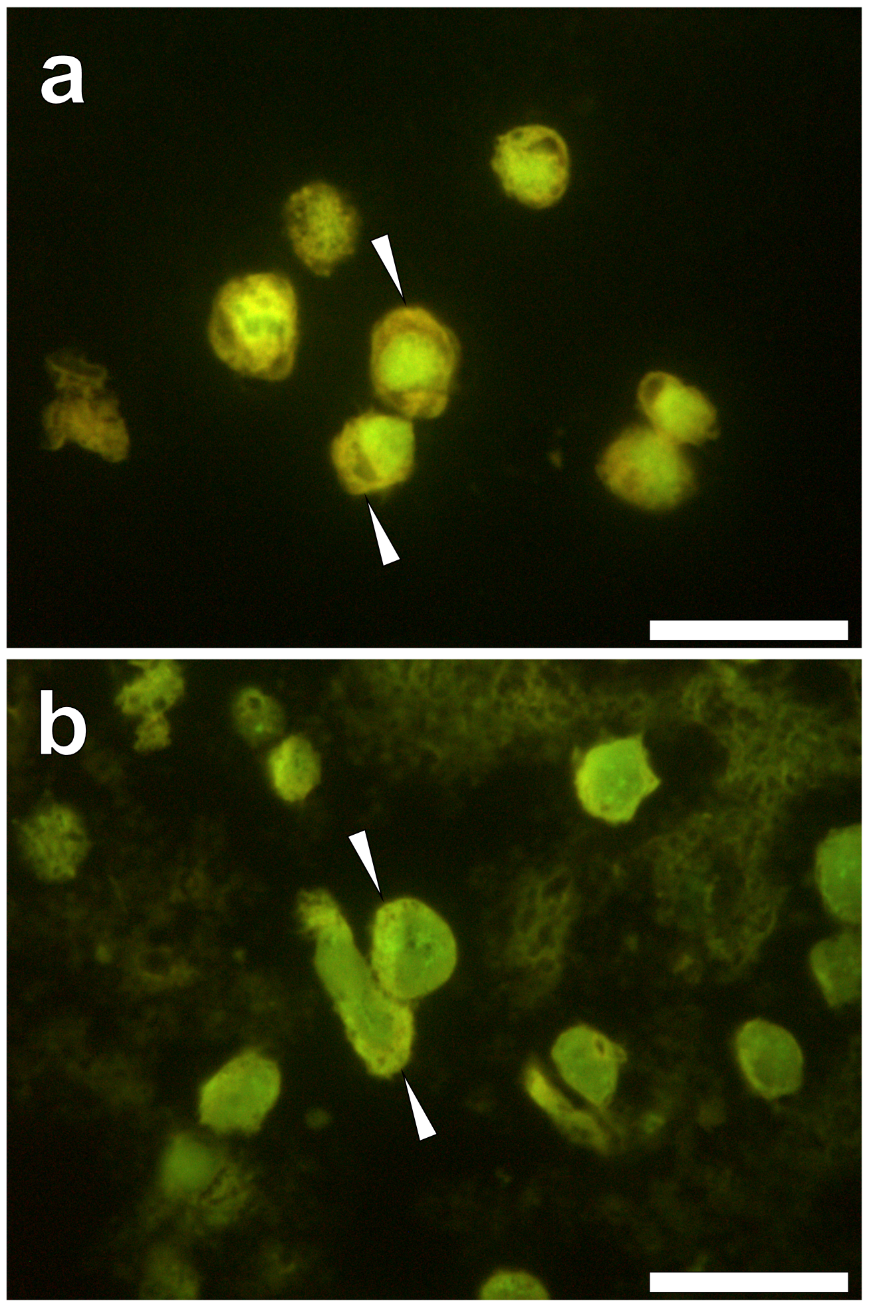
**

**Supplementary Fig. 2** Conventional (Shaw’s) morin staining of THP-1 cells co-cultured in the absence **(a)** and presence **(b)** of 50μg/mL Alhydrogel^®^. Sectioned THP-1 cells (5μm) were de-waxed, rehydrated and incubated with PBS for 10 min. Sections were subsequently incubated in 1% aqueous HCl for 10 min and rinsed twice in ultrapure water for 5 min, prior to staining in 0.2% *w/v* morin in 85% *v/v* ethanol containing 0.5% *v/v* acetic acid for 10 min. Sections were subsequently twice rinsed in ultrapure water for 5 min, dehydrated through 70, 90 and 100% *v/v* ethanol, cleared in xylene and mounted with Omnimount (National Diganostics™). Slides were viewed under a U-MNIB3 fluorescence filter cube (longpass λ_em_: 510nm) for morin fluorescence (green). Arrows depict yellow cytoplasmic fluorescence in both the absence **(a)** and presence **(b)** of Alhydrogel^®^. Scale bars: 20μm.

**Heightened autofluorescence for EDTA-morin versus lumogallion stained native T helper 1 (THP-1) cells, prepared in the absence of added adjuvant.**

**
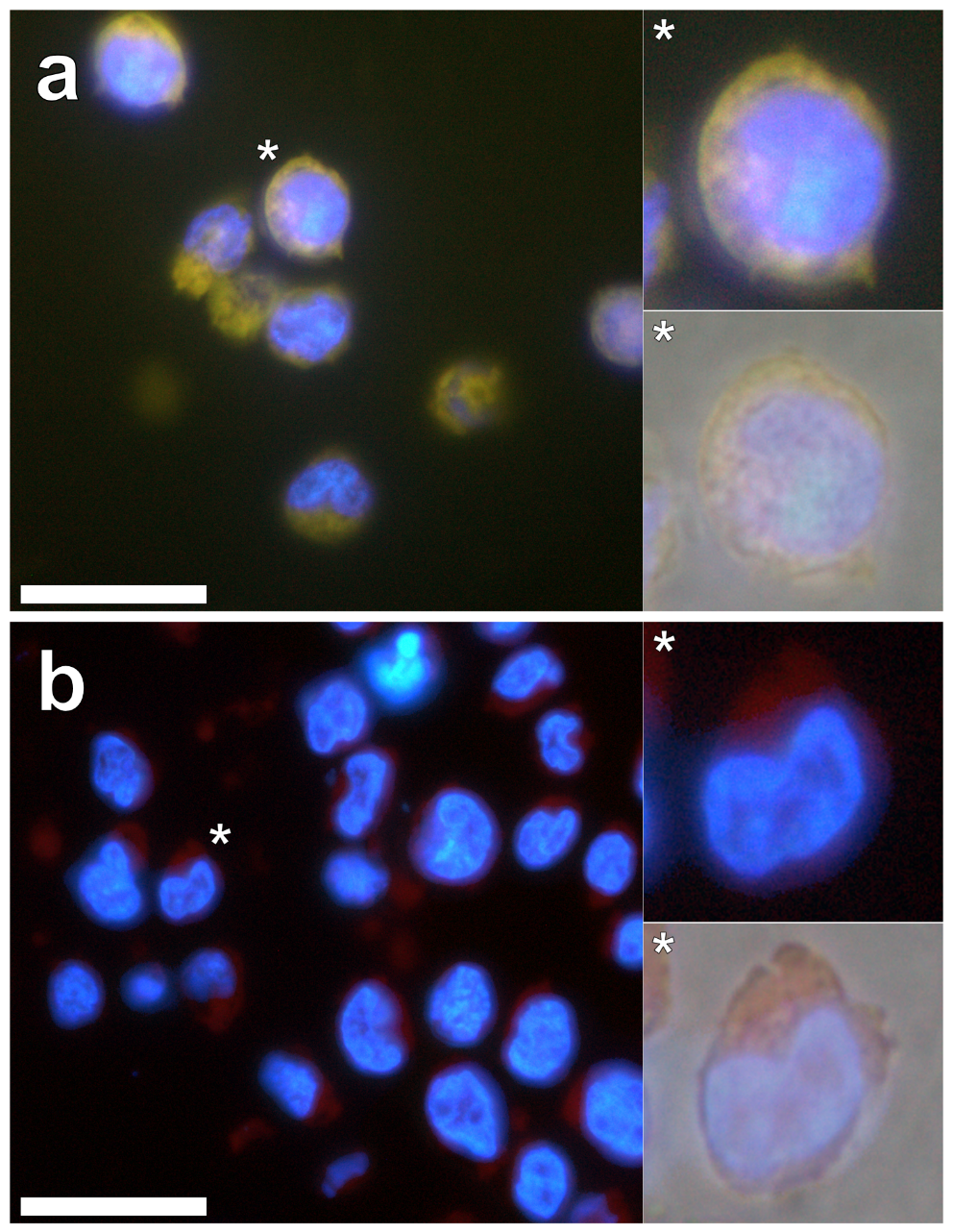
**

**Supplementary Fig. 3** Native THP-1 cell sections (5μm) prepared in the absence of added adjuvant and stained with lumogallion and EDTA-morin. **(a)** Morin stained sections were incubated in 5mM Na_2_EDTA for 10 min, rinsed for 35s in ultrapure, stained with 0.2% *w/v* morin in 85% *v/v* ethanol for 10 min, rinsed in 85% *v/v* ethanol and washed for 30s in ultrapure water and visualised under a U-MNIB3 (Olympus, UK) fluorescence filter cube (longpass λ_em_: 510nm). Lumogallion staining **(b)** was performed using 1mM of the fluorophore with staining performed over 45 min and visualised by use of a U-MNIB3 (Olympus, UK) fluorescence filter cube (single bandpass λ_em_: 570 – 610nm). All sections were mounted with ProLong Gold antifade reagent with DAPI (Fisher Scientific, UK) and viewed under a U-MWU2 cube (longpass λ_em_: 420nm) for DAPI staining of cell nuclei (blue). Scale bars: 20μm.

**Sudan Black B (SBB) quenching of lipofuscin fluorescence in the visualisation of aluminium in human brain tissue sections.**

**
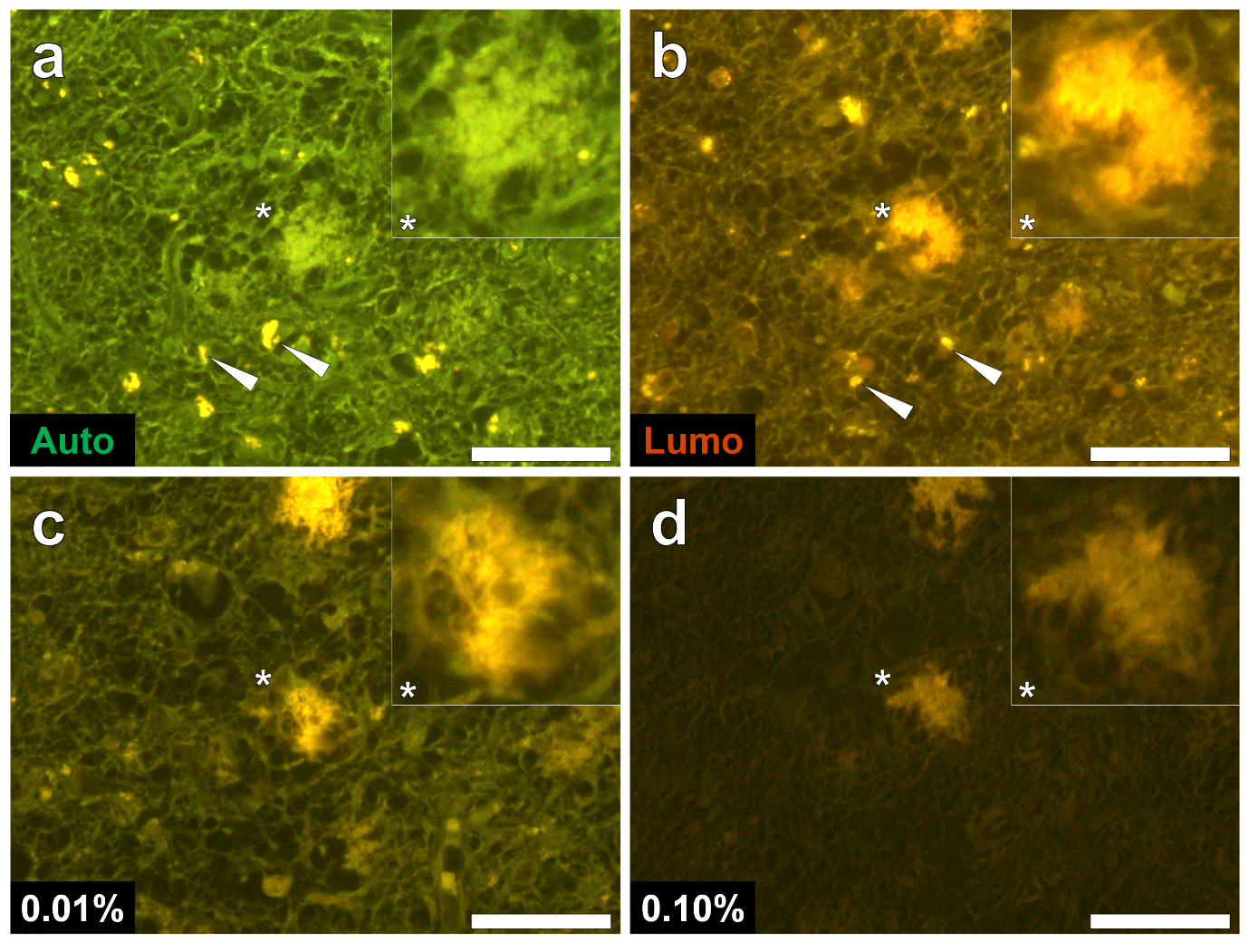
**

**Supplementary Fig. 4** Lumogallion staining and post Sudan Black B (SBB) induced-quenching of lipofuscin (white arrows), deposited in adjacent serial sections (5μm) of the parietal cortex of a 65 year old female donor (A8) with familial Alzheimer’s disease (fAD). **(a)** Autofluorescence upon staining (45 min) in 50mM PIPES buffer, pH 7.4, only. **(b)** Lumogallion fluorescence (orange) of a senile plaque (asterisk) contrasted against lipofuscin fluorescence (yellow) upon staining with 1mM of the fluorophore in the same PIPES buffer. **(c)** 0.01% *w/v* post-SBB staining (10 min, 70% *v/v* ethanol) of lumogallion stained tissue **(d)** 0.10% *w/v* post-SBB stained tissue. An aluminium-reactive senile plaque (asterisks) is highlighted across all adjacent serial sections, of which lipofuscin fluorescence was quenched upon post-staining with SBB. All sections were viewed under a U-MNIB3 (Olympus, UK) fluorescence filter cube. Scale bars: 50μm.
